# Diagnostic value of urinary exosomes in patients with IgA nephropathy and diabetic kidney disease: a systematic review and meta-analysis

**DOI:** 10.3389/fmed.2026.1811135

**Published:** 2026-07-23

**Authors:** Ru Xu, Lele Jiang

**Affiliations:** Department of Clinical Laboratory, Beilun People's Hospital, Ningbo, China

**Keywords:** diabetic kidney disease (DKD), diagnosis, IgA nephropathy (IgAN), meta-analysis, urinary exosomes

## Abstract

**Background:**

Previous studies have indicated an association between the novel biomarker urinary exosomes and both IgA nephropathy (IgAN) and diabetic kidney disease (DKD). This study aimed to investigate the diagnostic value of urinary exosomes in patients with IgAN and DKD.

**Methods:**

The PubMed, Embase, Cochrane Library, and Web of Science databases were searched to identify eligible studies from their inception to December 4, 2025. Data on sensitivity, specificity, positive likelihood ratio (PLR), negative likelihood ratio (NLR), diagnostic odds ratio (DOR), and their corresponding 95% confidence intervals (95% CI) were extracted and pooled. Diagnostic meta-analysis was performed using Stata software (version 15.0; Stata Corporation, College Station, TX, USA) and Meta-DiSc version 1.4.

**Results:**

A total of 22 articles involving 6,482 participants were included. The pooled results showed that for the diagnosis of DKD, urinary exosomes had a sensitivity of 0.86 (95% CI: 0.77–0.92), a specificity of 0.83 (95% CI: 0.75–0.89), a DOR of 31.10 (95% CI: 11.09–87.19), and an area under the curve (AUC) of 0.91. For the diagnosis of IgAN, urinary exosomes had a sensitivity of 0.78 (95% CI: 0.71–0.83), a specificity of 0.70 (95% CI: 0.63–0.77), a DOR of 8.26 (95% CI: 5.03–13.56), and an AUC of 0.81.

**Conclusion:**

Urinary exosomes showed stronger summary diagnostic performance for DKD than for IgAN. For IgAN, the pooled results support a moderate diagnostic contribution rather than a clearly established clinical role. Standardized prospective studies with prespecified thresholds are needed before routine clinical use can be established.

**Systematic review registration:**

identifier: CRD420251252373.

## 1 Introduction

Chronic kidney disease (CKD) has become a major global public health issue threatening human health (1). It is characterized by an insidious onset and slow progression, potentially advancing to end-stage renal disease (ESRD) in its late stages. In 2023, it was estimated that 788 million (95% CI: 743–843) individuals aged 20 years and older were affected by CKD (2), imposing a substantial medical and economic burden on patients, their families, and society. IgA nephropathy (IgAN), the most common primary glomerulonephritis worldwide, is a significant cause of CKD (3). Diabetic kidney disease (DKD) is a leading cause of both CKD and ESRD globally.

The gold standard for diagnosing IgAN and DKD is renal biopsy (4). However, this procedure is invasive, and dynamic monitoring of disease progression requires repeated operations, which carries risks of trauma, infection, and bleeding (5). Furthermore, traditional blood and urine biomarkers (such as serum creatinine, blood urea nitrogen, and urinary protein) lack sufficient sensitivity in the early stages of kidney damage, thus failing to meet the clinical need for early diagnosis and disease assessment of these two conditions. This often leads to delayed diagnosis and missed optimal intervention windows. Therefore, there is a clinical demand for novel, non-invasive biomarkers with high sensitivity and specificity to improve the early diagnosis of patients with IgAN and DKD. Urinary exosomes, as a form of non-invasive liquid biopsy (6), are extracellular vesicles secreted by renal cells. These vesicles contain various biomolecules, such as proteins, RNA, and DNA. These biomolecules can reflect the unique physiological and pathological states of the kidney (7) and, due to their good stability in urine, are considered to have potential application value in the early diagnosis, disease monitoring, and progression prediction of kidney diseases.

Currently, no systematic review or meta-analysis has comprehensively evaluated the diagnostic accuracy of urinary exosomes for IgAN and DKD or quantitatively synthesized the diagnostic performance, including sensitivity, specificity, and the area under the receiver operating characteristic curve of different exosomal biomarkers. Consequently, this study systematically assesses the diagnostic efficacy of urinary exosomes for IgAN and DKD through a systematic review and meta-analysis that synthesizes existing evidence. This work aims to provide an evidence-based foundation for developing non-invasive diagnostic strategies for these renal conditions.

## 2 Methods

This systematic review and meta-analysis was conducted in accordance with the Preferred Reporting Items for Systematic Reviews and Meta-Analyses for Diagnostic Test Accuracy Studies (PRISMA-DTA) (8). The study protocol was registered on the PROSPERO platform (Registration Number: CRD420251252373).

### 2.1 Search strategy

A comprehensive literature search was conducted across the PubMed, Embase, Cochrane Library, and Web of Science databases. Publications were limited to those in English and published from each database's inception until December 4, 2025. The search strategy combined Medical Subject Headings (MeSH) terms with free-text keywords. The specific MeSH terms and keywords included Glomerulonephritis, IGA; Diabetic Nephropathy; exosome; and diagnosis (Supplementary Text 1).

### 2.2 Literature screening

**Inclusion criteria**: (1) Study population: Patients suspected of having IgAN or DKD; (2) Index test: Any biomarker (including but not limited to miRNA, protein, or other molecules) in urinary exosomes; (3) Reference standard: Renal histopathological examination, or diagnosis based on established clinical diagnostic criteria with exclusion of other types of kidney disease; (4) Outcome: The study must provide, or allow calculation from raw data, the complete data required for assessing diagnostic accuracy, including sensitivity (SEN), specificity (SPE), true positives (TP), false positives (FP), false negatives (FN), and true negatives (TN); (5) Study design: Cohort studies, cross-sectional studies, and case-control studies.

**Exclusion criteria**: (1) Reviews, meta-analyses, conference abstracts, editorials, letters, replies, case reports, commentaries, brief surveys, or notes; (2) Clinical trials, animal or *ex vivo* studies; (3) Duplicate publications and literature for which full text was unavailable; (4) Literature from which outcome measures could not be extracted; (5) Other specified conditions.

Two reviewers, XR and JLL, independently screened the literature according to the aforementioned criteria. Any disagreements encountered during the study selection process were resolved through discussion or by consultation with a third reviewer, LCY.

### 2.3 Data extraction and quality assessment

Two reviewers (XR and JLL) independently extracted data from the finally included studies. The extracted information included the first author, publication year, country, basic characteristics of the study population, detection method, detection index, cut-off value, SEN, SPE, TP, FP, FN, and TN. These data were either directly provided or could be calculated from the original data.

The included studies were independently assessed for quality and applicability by two reviewers (XR and JLL) using the QUADAS-2 (Quality Assessment of Diagnostic Accuracy Studies 2) tool (9). This tool comprises four domains for assessing risk of bias (patient selection, index test, reference standard, and flow and timing) and three domains for assessing applicability (patient selection, index test, and reference standard). Within each domain, the risk of bias is judged as ‘low' if all signaling questions are answered ‘yes', as ‘high' if any signaling question is answered ‘no', and as ‘unclear' when information is insufficient. Any disagreements between the reviewers were resolved through discussion to reach a consensus. If consensus cannot be achieved, a third reviewer (LLX) made the final judgment. The methodological quality of the included studies was documented and graphically presented using RevMan software (version 5.4).

### 2.4 Data integration and statistical analysis

Diagnostic meta-analysis was performed using Stata software (version 15.0; Stata Corporation, College Station, TX, USA) and Meta-DiSc version 1.4. A bivariate generalized linear mixed-effects model was applied to calculate the pooled sensitivity, specificity, positive likelihood ratio (PLR), negative likelihood ratio (NLR), diagnostic odds ratio (DOR), and their corresponding 95% confidence intervals (95% CIs). A summary receiver operating characteristic (SROC) curve was constructed, and the area under the curve (AUC) was calculated to assess the overall diagnostic accuracy. The presence of a threshold effect was evaluated using the Spearman correlation coefficient between sensitivity and specificity, along with the corresponding *P* value. Because the included studies reported study-specific cut-off values, no common threshold was assumed across studies. The bivariate model was used to pool sensitivity and specificity while allowing for between-study threshold variation. When *P* > 0.05, heterogeneity attributable to threshold effects was considered not significant. Subsequently, Cochran's Q test and the *I*^2^ statistic were used to assess heterogeneity among the included studies. Significant heterogeneity was defined as *P* < 0.10 and *I*^2^ > 50%. To explore potential sources of heterogeneity, subgroup analyses and meta-regression analyses were conducted. Finally, Deeks' funnel plot asymmetry test was performed to evaluate publication bias, with *P* > 0.05 indicating no significant publication bias.

## 3 Results

### 3.1 Workflow

**Figure 1:**
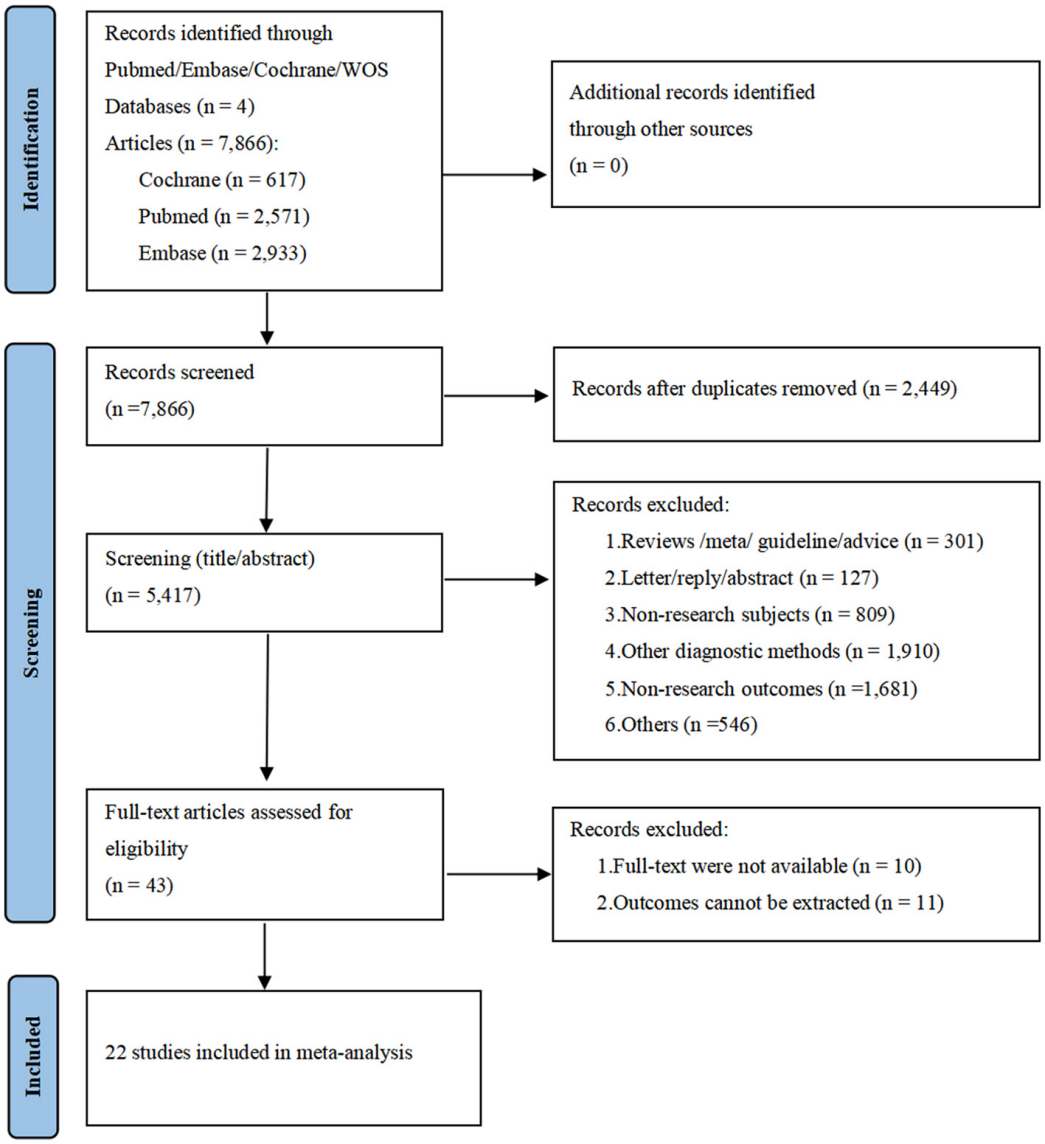
The PRISMA flowchart of the literature search and selection.

A total of 7,866 articles were initially retrieved from the database search, and no further studies were identified through reference scanning. After duplicate removal, 5,417 articles were screened by title and abstract. Of these, 4,374 articles were excluded for not meeting the inclusion criteria, while 43 articles underwent a detailed full-text review. Ultimately, 22 studies (10–31) were included in this meta-analysis (Figure 1).

### 3.2 Study characteristics

A total of 22 studies from 5 countries (China, Japan, the United Kingdom, Macedonia, and Korea) were included, involving 2,956 patients. The age of the enrolled patients spanned various age groups. The publication years ranged from 2011 to 2025. The study designs were heterogeneous, with most studies using case-control designs and a smaller number using cohort or cross-sectional designs. The detection methods were primarily based on molecular biology techniques, alongside various other technologies such as ELISA, Western blot, TEM/NTA/MS. The biomarkers were predominantly microRNAs (miRNAs), and also included protein-based biomarkers such as Aminopeptidase N and Vasorin precursor (Table 1).

**Table 1:** Study characteristic.

No	References	Country	Study design	*N* (male/female)	Age Mean ± SD or Median (IQR) or Median [range] or range	Disease type	Control type	Urine exosomal biomarker	Detection method	Reference standard	Main index	Exosome isolation methods
1	Szeto CC et al. (30)	China	cohort	IgAN:33(10/23) healthy control:9(4/5)	IgAN: 45.1 ± 10.2 healthy control: 37.4 ± 4.0	IgAN	Healthy controls	miR−150, miR−204, miR−431, miR−55	RT–QPCR	renal biopsy	ROC/AUC/ miR−204 of Sensitivity/ Specificity	Not application
3	Li T et al. (26)	China	cross–sectional	DN: 48 (28/20) T2DM: 48 (28/20)	DN: 61 (51.25–66.75) T2DM: 58.0 (49.5–63.0)	DN	T2DM	PAK 6, EGFR, PAK 6+EGFR	TEM/NTA/ MS/Western blotting	the guidelines of American Diabetes Association	ROC/AUC	Size exclusion chromatography methods
4	Duan Z–Y et al. (20)	China	cohort	Confirmation cohort: IgAN patients:30 (18/12) Normal control:20 (10/10) Validation cohort: IgAN patients: 63 (33/30) Normal control:62 (34/28) Disease control:40 (16/24)	Confirmation cohort: IgAN patients:34.37 ± 10.334 Normal control: 34.37 ± 10.334 Validation cohor: IgAN patients:34.92 ± 10.338 Normalcontrol: 34.21 ± 7.41 Disease control: 43.07 ± 17.457	IgAN	Normal control, Disease control	miR−25–3p, miR−144–3p, miR−486–5p	qRT–PCR	renal biopsy	ROC/AUC/ Sensitivity/ Specificity	Not application
5	Zou L–X et al. (17)	China	cohort	DKD: 121 (71/50) DM: 227 (138/89)	DKD: 55.0 (47.0; 65.0) DM: 55.0 (47.0; 65.0)	DKD	DM	cross–sectional: ntOPN, NGAL, MCP−1	competitive enzyme–linked immunosorbent assays (ELISAs)	the International Conference on Harmonization guidelines for Good Clinical Practice	ROC/AUC/ Sensitivity/ Specificity	Not application
6	Zheng S et al. (14)	China	cohort	DKD: 19 (12/7) DM: 37 (29/8)	DKD: 60.53 ± 11.97 DM: 53.05 ± 11.96	DKD	DM	miR−136–5 p	Western blot/qRT–PCR	2020 ADA Standards, urinary albumin/creatinine ratio (UACR)>300 mg/g or confirmed by renal biopsy	ROC/AUC/ Sensitivity/ Specificity /PPV/NPV	Ultracentrifugation
7	Zhang Q et al. (23)	China	cohort	IgAN: 40 (22/18) Non–IgAN: 30 (17/13) healthy control: 21 (12/9)	IgAN: 41.2 ± 12.3 Non–IgAN: 42.1 ± 15.2 healthy control: 38.1 ± 10.5	IgAN	Non–IgAN, healthy control	miR−451a	qRT–PCR	renal biopsy	ROC/AUC	Ultracentrifugation
8	Zhang M et al. (29)	China	cohort	Confirmation cohort: IgAN patients: 30 (17/13) Normal control: 30 (17/13) Validation cohort: IgAN: 144 (78/66) disease control: 100 (55/45) healthy control: 67 (33/34)	Confirmation cohort: IgAN patients: 36.30 ± 9.75 Normal control:36.92 ± 10.27 Validation cohort: IgAN:36.51 ± 10.42 disease control: 43.72 ± 14.19 healthy control:38.50 ± 9.16	IgAN	disease control, healthy control	miR−16–5p, Let−7g−5p, miR−15a−5p	qRT–PCR	renal biopsy	ROC/AUC/ Sensitivity/ Specificity/ PPV/NPV	Not application
9	Zhang H et al. (11)	China	cohort	HC: 81 (43/38) DM: 187 (149/38) DN: 132 (94/38) NDRD: 163 (125/38) NDRD–DM: 86 (46/40)	HC: 49.80 (14.47) DM: 55.22 (11.85) DN: 58.76 (13.53) NDRD: 46.39 (15.38) NDRD–DM: 49.48 (14.53)	DN	HC, DM, NDRD, NDRD–DM	CKAP4	qRT–PCR	renal biopsy	ROC/AUC/ Sensitivity/ Specificity	WGA–coupled magnetic beads isolation of urinary extracellular vesicles (uEVs)
11	Wang J et al. (15)	China	cross–sectional	DKD: 42 (28/14) T2DM: 21 (10/11)	DKD: 61.57 ± 11.90 T2DM: 55.33 ± 13.33	DKD	T2DM	miRNA−615–3p, miRNA−3147, miRNA−615– 3 p??Cystatin C, TGF–β 1?ACR	RT–qPCR	Guidelines published in 2010 by the American Diabetes Association were employed as the diagnostic criteria for T2DM patients, and The National Kidney Foundation Kidney Disease Outcomes Quality Initiative (NKFKDOQI) guidelines were used as the diagnostic criteria for selecting patients with DKD	ROC/AUC	High–speed centrifugation + ultrafiltration enrichment + ExoQuickTM Exosome Precipitation Kit
12	Li S et al. (13)	China	cohort	DN: 54 (36/18) T2DM: 39 (30/9)	DN: 59.57 ± 11.59 T2DM: 52.67 ± 12.10	DN	T2DM	miR−142–3p	TEM/NTA/Western blotting/qRT–PCR	Diagnostic criteria from the Standards of Medical Care in Diabetes (2020) issued by the American Diabetes Association (ADA), accompanied by renal biopsy pathology results or a urinary albumin–to–creatinine ratio (UACR) > 30 mg/g, with the UACR reaching or exceeding the cutoff value twice within 3–6 months after repeated tests.	AUC/ Sensitivit/ Specificity/ Positive predictive/ Negative predictive	Ultracentrifugation
13	Li S et al. (22)	China	cohort	Validation cohorts: IgAN: 20 (16/4) HC: 20 (12/8)	Validation cohorts: IgAN37.65 ± 12.01 HC: 45.5 ± 14.17	IgAN	HC	hsa–miR−451a, hsa–mir−7d−3p, hsa–miR−451 a+hsa–let−7 d−3 p	qRT–PCR	renal biopsy	ROC/AUC/ sensitivity/ specificity	Urine exosome extraction kit
14	Zhao Y et al. (24)	China	case–control	Validation cohorts: DM: 14 (7/7) DKD: 14 (7/7)	Validation cohorts: DM:57.79 ± 8.51 DKD: 57.79 ± 8.51	DKD	DM	miR−4534	qRT–PCR	All enrolled patients with diabetic kidney disease (DKD) met the above–mentioned WHO diagnostic criteria for diabetes, and were complicated with overt proteinuria (24–h urinary microalbumin ≥ 300 mg/24 h) and retinopathy.	AUC/ sensitivity/ specificity	Exosome Isolation Kit
18	Abe H et al. (25)	Japan	cross–sectional	DN (*n* = 10) MCNS (*n* = 10)	DN: 57.0 ± 6.2 MCNS: 32.8 ± 15.1	DN	MCNS	WT1 mRNA	qRT–PCR	renal biopsy	ROC/AUC	Ultracentrifugation + sucrose density gradient ultracentrifugation
19	Bai X et al. (12)	China	cross–sectional	DN: 71 (37/34) Diabetes: 74 (38/36)	DN: ≤ 68 31 (43.66) >68 40 (56.34) Diabetes: ≤ 68 32 (43.24) >68 42 (56.76)	DN	Diabetes	VDBP	ELISA	renal biopsy	ROC/AUC	Not application
20	Beltrami C et al. (10)	United Kingdom	cross–sectional	Diabetic: 62 (37/25) DKD: 89 (55/34) Controls: 41 (18/23)	Diabetic: 52 ± 16.1 DKD: 62 ± 13.6 Controls: 55 ± 15.4	DKD	Diabetes, Controls	miR−126, miR−155, miR−29b, All Three miRNAs	RT–qPCR	National Kidney Foundation–Kidney Disease Outcomes Quality Initiative (NKF–KDOQI) Clinical Practice Guidelines and Clinical Practice Recommendations for Diabetes and Chronic Kidney Disease (CKD)	Sensitivity/ Specificity/ Likelihood ratio/ROC/ AUC	Not application
21	Ding X et al. (23)	China	cross–sectional	DN: 8 (5/3) NDRD: 5 (3/2) T2DM: 7 (4/3) HC: 9 (5/4)	DN: 57.50 ± 7.71 NDRD: 58.00 ± 5.00 T2DM: 54.29 ± 11.64 HC: 47.67 ± 6.00	DN	NDRD, T2DM, HC	PHYHD 1	ELISA	renal biopsy	ROC/AUC/ sensitivity/ specificity	Ultracentrifugation combined with 0.22 μm filtration
22	Feng Y et al. (27)	China	cross–sectional	IgAN: 55 (31/24) Healthy Controls: 24 (11/13)	IgAN: 38.6 ± 13.3 Healthy Controls: 38.2 ± 13.8	IgAN	HC	CCL 2	RT–qPCR	renal biopsy	ROC/AUC	Differential centrifugation + ultracentrifugation
23	Han L–L et al. (21)	China	cross–sectional	NC: 20 (8/12) DM: 20 (11/9) DKD: 20 (13/7)	NC: 45.80 ± 14.69 DM: 50.15 ± 13.50 DKD: 48.79 ± 15.14	DKD	DM, NC	miR−145–5p, miR−27a−3p, miR−145– 5 p+miR 27 a−3 p	RT–qPCR	ADA	ROC/AUC/sensitivity/specificity	Centrifugation at 3,000 × g for 20 min at 4 °C + 0.22 μm filtration
24	Kostovska I et al. (31)	Macedonia	cross–sectional	DN: 30 Diabetic: 60 Healthy subjects: 30	DN: 55.8 ± 10.1 Diabetic: 57.9 ± 6.7 Healthy subjects: 47.8 ± 9.3	DN	Diabetic, Healthy subjects	nephrin	indirect competitive ELISA	The Department of Nephrology, Faculty of Medicine, Ss. Cyril and Methodius University in Skopje recruited patients diagnosed with diabetic nephropathy (DN), with the inclusion criteria as follows: the nephropathy patients were those with type 2 diabetes mellitus (T2DM) and clinically diagnosed nephropathy, defined as macroalbuminuria alone or combined with macroalbuminuria and renal dysfunction.	Accuracy/ROC	Not application
25	Li WT et al. (18)	China	cross–sectional	Healthy controls: 15 (5/10) Diabetes: 20 (13/7) DN: 28 (14/14)	Healthy controls: 46 ± 11.78 Diabetes: 53.8 ± 15.57 DN: 54.89 ± 13.76	DN	DM, NC	hsa–let−7c−5p, hsa–miR−29c−5p, hsa–miR−15b−5p	RT–PCR	renal biopsy	ROC/AUC/ sensitivity/ specificity	Differential centrifugation + ultracentrifugation
29	Moon PG et al. (19)	Korea	cross–sectional	Normal: 6(6/0) IgAN: 12 (9/3) TBMN: 12 (11/1)	Normal: 18.83 ± 0.41 IgAN: 31 ± 14.37 TBMN: 32.33 ± 15.15	IgAN	Normal, TBMN	Aminopeptidase N/Vasorin precursor/a−1–antitrypsin/Ceruloplasmin	Western blot	renal biopsy	ROC/AUC/ sensitivity/ specificity	Ultracentrifugation + DTT treatment + sucrose density gradient ultracentrifugation
32	Szeto CC et al. (28)	China	cross–sectional	IgAN: 72 (33/39) HTN: 34 (22/12) CTL: 20 (11/9)	IgAN: 47.2 ± 15.7 HTN: 56.6 ± 17.5 CTL: 44.3 ± 17.3	IgAN	HTN, CTL	miR−106a, miR−145, miR−196b, miR−1248, miR−345, miR−363	NanoString miRNA assay	renal biopsy	ROC/AUC/ sensitivity/ specificity	Not application

### 3.3 Quality assessment

**Figure 2:**
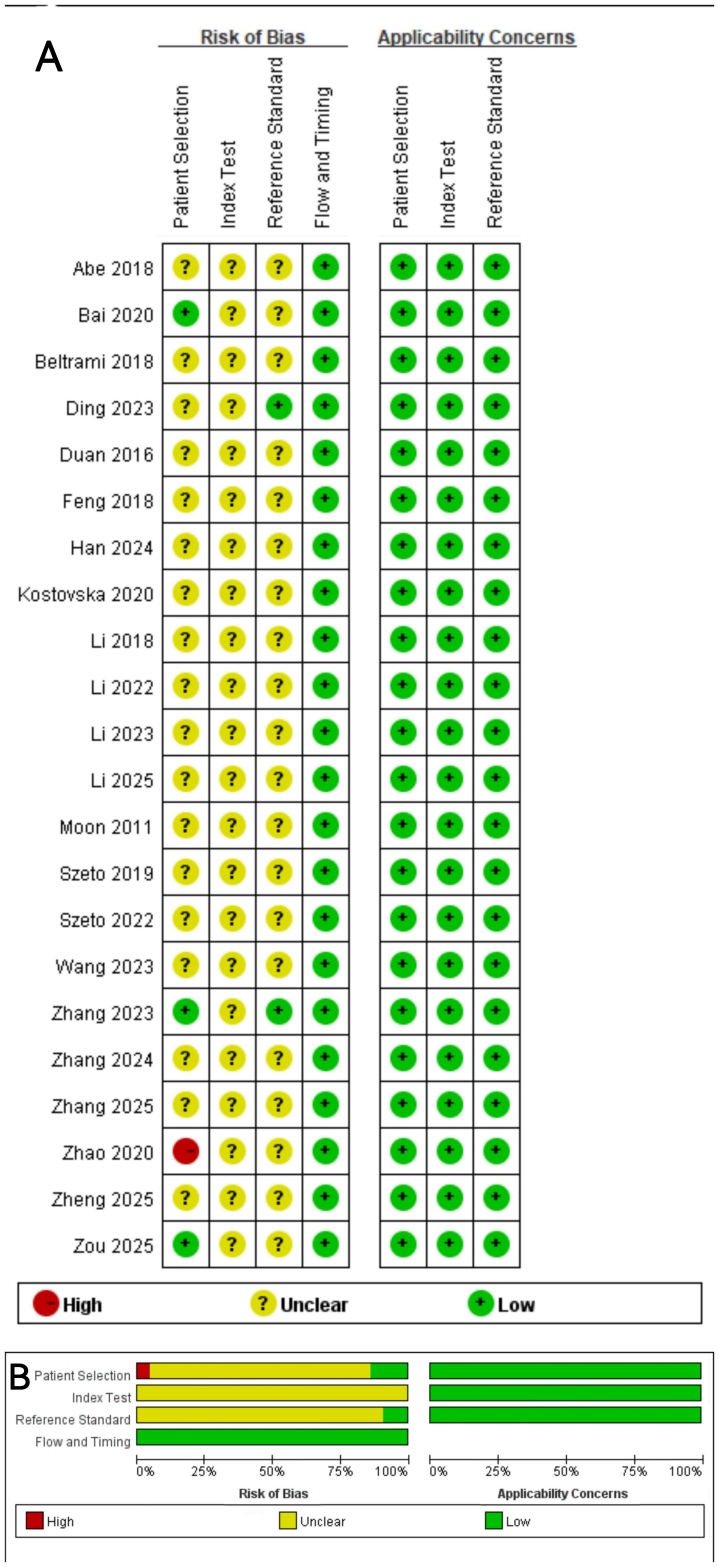
Quality assessment for 22 studies (Quality Assessment of Diagnostic Accuracy Studies [QUADAS]−2). **(A)** Risk of bias and applicability concerns for each included study, classified as low, unclear, or high. **(B)** Summary bar chart showing the proportion of studies judged as having low, unclear, or high risk of bias and applicability concerns across all included studies.

Summary of Risk of Bias and Applicability Concerns (Figure 2). Patient selection was the primary source of bias risk, with the vast majority of studies rated as “unclear” (yellow), only a few as “low” (green), and one as “high” (red). This is directly related to the design characteristics of the included case-control studies. The risk of bias regarding the reference standard was mostly unclear, primarily because most studies did not report whether blinding to urinary exosome results was performed during pathological assessment. The risk of bias for the index test was also generally “unclear,” reflecting insufficient reporting of relevant details. In contrast, the risk of bias in the flow and timing domain was rated as “low risk” (green) for all studies, indicating standardized procedures in this aspect. All applicability concerns were judged as low risk. Still, the frequent unclear or high risk of bias in patient selection and reference standard, together with the predominance of case-control designs, may have inflated the pooled diagnostic estimates and limit direct transfer of these findings to routine clinical settings.

### 3.4 Meta-analysis

**Figure 3:**
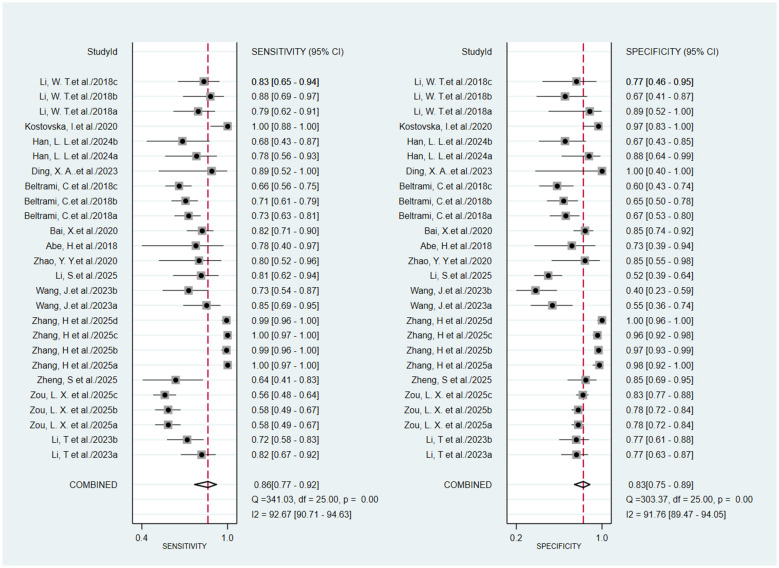
Forest plot of sensitivity and specificity of urinary exosomes in the diagnosis of diabetic kidney disease (DKD) across all included studies.

**Figure 4:**
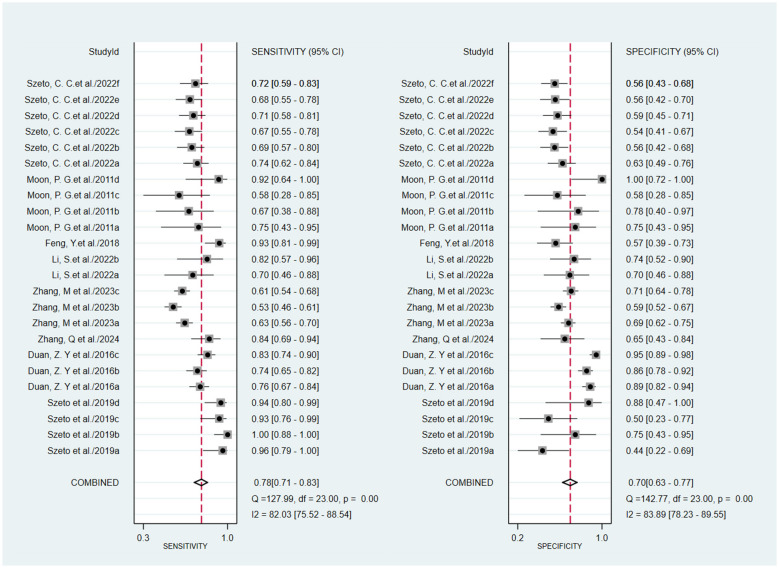
Forest plot of sensitivity and specificity of urinary exosomes in the diagnosis of IgAN across all included studies.

**Figure 5:**
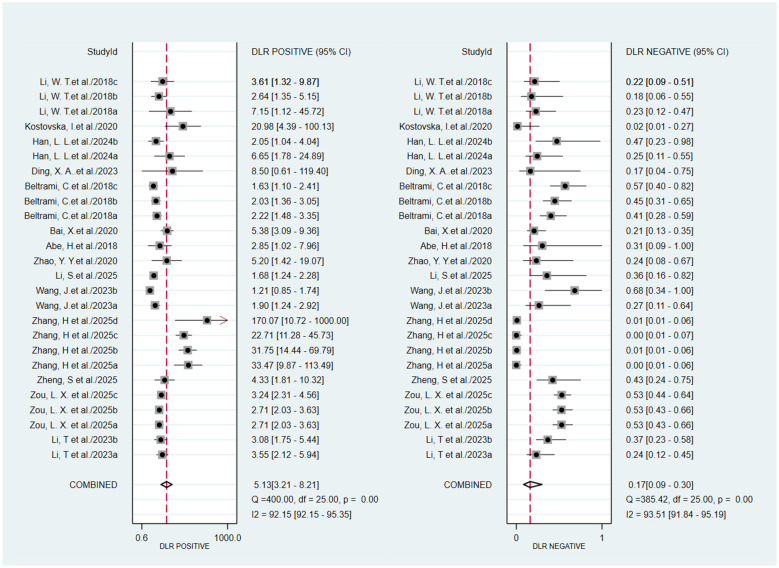
Forest plot of DLR POSITIVE and DLR NEGATIVE of urinary exosomes in the diagnosis of diabetic kidney disease (DKD) across all included studies.

**Figure 6:**
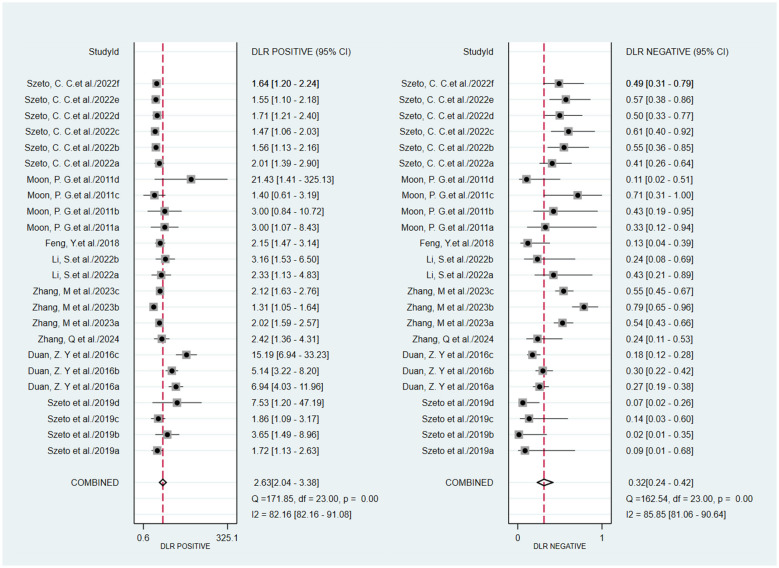
Forest plot of DLR POSITIVE and DLR NEGATIVE of urinary exosomes in the diagnosis of IgAN across all included studies.

**Figure 7:**
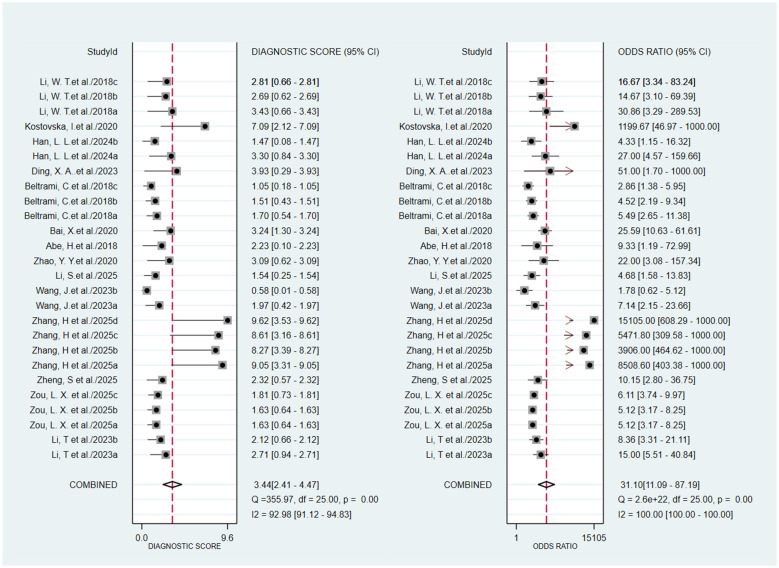
Forest plot of DOR of urinary exosomes in the diagnosis of diabetic kidney disease (DKD) across all included studies.

**Figure 8:**
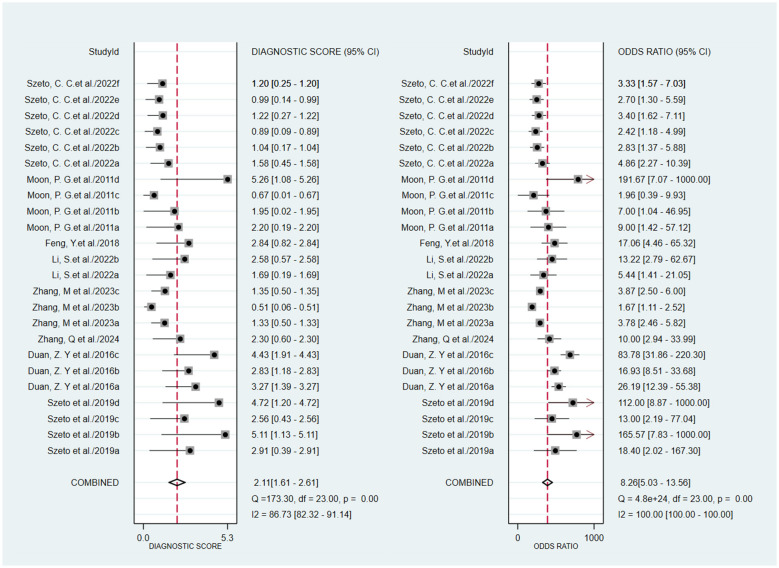
Forest plot of DOR of urinary exosomes in the diagnosis of IgAN across all included studies.

**Figure 9:**
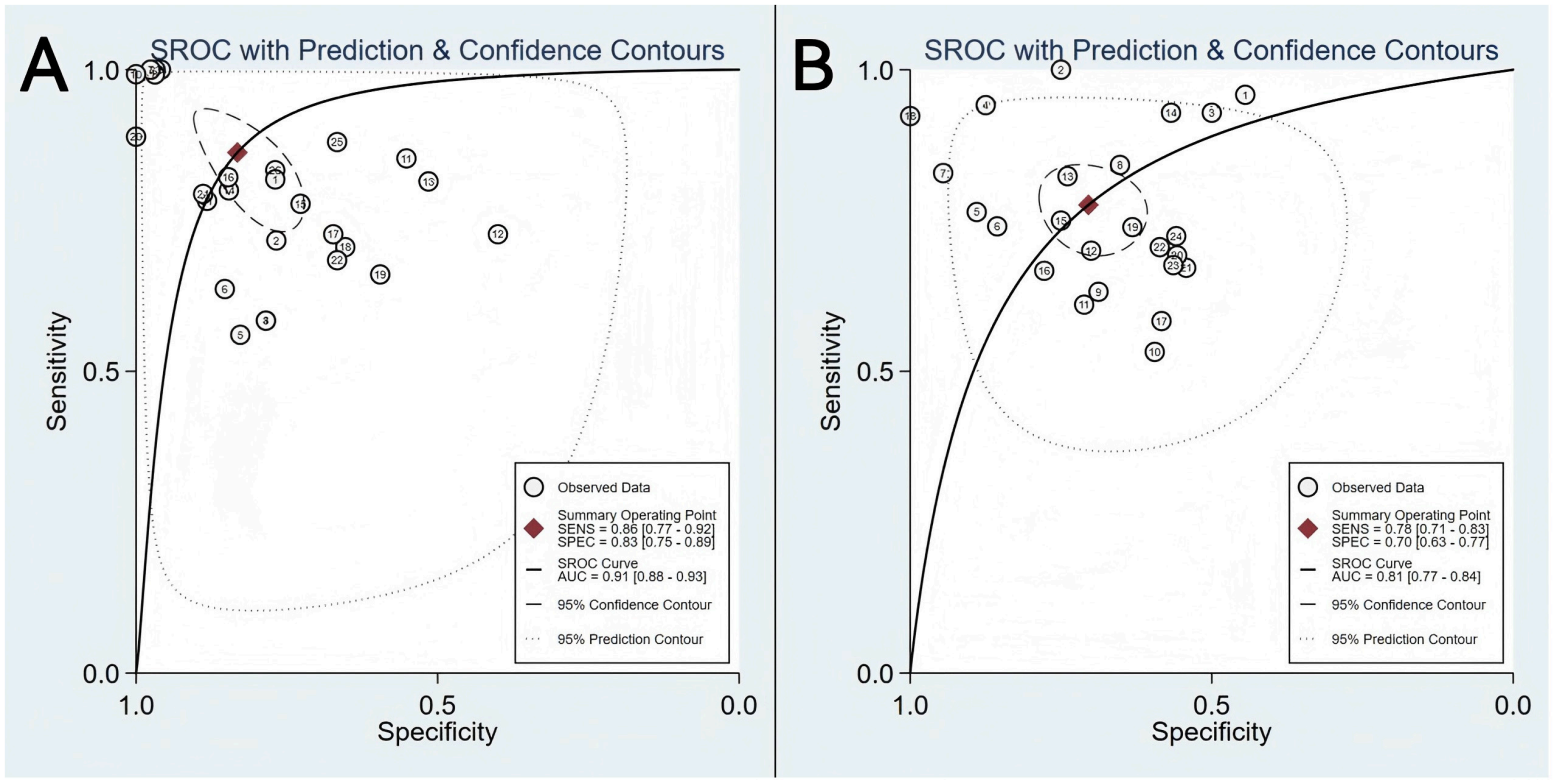
**(A)** ROC curve of urinary exosomes for DKD diagnosis across all included studies. **(B)** ROC curve of urinary exosomes for IgAN diagnosis across all included studies.

For DKD, the pooled diagnostic SEN was 0.86 (95% CI: 0.77–0.92) and SPE was 0.83 (95% CI: 0.75–0.89) (Figure 3). The corresponding pooled estimates for IgAN were a SEN of 0.78 (95% CI: 0.71–0.83) and a SPE of 0.70 (95% CI: 0.63–0.77) (Figure 4). The PLR for DKD was 5.13 (95% CI: 3.21–8.21), with a NLR of 0.17 (95% CI: 0.09–0.30) (Figure 5). For IgAN, the pooled PLR was 2.63 (95% CI: 2.04–3.38) and the NLR was 0.32 (95% CI: 0.24–0.42) (Figure 6). Analysis of the DOR yielded a pooled value of 31.10 (95% CI: 11.09–87.19) for DKD (Figure 7) and 8.26 (95% CI: 5.03–13.56) for IgAN (Figure 8). In the SROC analysis, the AUC was 0.91 (95% CI: 0.88–0.93) for DKD and 0.81 (95% CI: 0.77–0.84) for IgAN (Figure 9). Threshold effect analysis revealed a significant threshold effect for DKD, with a Spearman correlation coefficient of−0.462 (*P* = 0.018), indicating heterogeneity related to study-specific cut-off values. No common diagnostic threshold was assumed across the included DKD studies; therefore, the pooled DKD estimates represent summary measures across variable thresholds, not the performance of a single uniform clinical cut-off. For IgAN, the coefficient was−0.618 (*P* = 0.432), indicating no significant threshold effect.

#### 3.4.1 Subgroup analysis and regression analysis

Subgroup analyses based on different covariates were applied to explore potential sources of heterogeneity: Control type (Healthy vs. Non-healthy), Biomarker type (miRNA vs. protein/polypeptide), Sample size (< 50 vs. ≥ 50), and Publication year (2011–2018 vs. 2019–2025) (Tables 2, 3).

**Table 2:** Subgroup analysis of urinary exosomes for IgAN diagnosis.

Group	Category	Sensitivity (95% CI)	*P*1	Specificity (95% CI)	*P*2	PLR (95% CI)	NLR (95% CI)	DOR (95% CI)	AUC (95% CI)
Control type	Healthy	0.91 (0.84–0.95)	0.45	0.63 (0.54–0.71)	0.04	2.5 (2.0–3.1)	0.14 (0.08–0.26)	17 (9–34)	0.76 (0.72–0.79)
	No Healthy	0.69 (0.64–0.74)		0.73 (0.63–0.80)		2.5 (1.8–3.6)	0.42 (0.33–0.54)	6 (3–11)	0.75 (0.71–0.79)
Biomarker type	miRNA	0.76 (0.70–0.82)	0.04	0.70 (0.62–0.77)	0.18	2.5 (1.9–3.3)	0.34 (0.25–0.45)	7 (4–13)	0.80 (0.76–0.83)
	protein/polypeptide	0.81 (0.64–0.91)		0.73 (0.53–0.86)		3 (1.6–5.7)	0.26 (0.13–0.54)	11 (3–38)	0.84 (0.80–0.87)
Sample size	< 50	0.87 (0.76–0.94)	0.20	0.71 (0.59–0.80)	0.16	3 (2.1, 4.2)	0.18 (0.09–0.35)	17 (7–40)	0.83 (0.79–0.86)
	≥ 50	0.72 (0.66–0.76)		0.70 (0.61–0.77)		2.4 (1.7, 3.2)	0.41 (0.32–0.53)	6 (3–10)	0.76 (0.72–0.80)
Publication year	2011–2018	0.79 (0.73–0.84)	0.04	0.84 (0.72–0.91)	0.79	4.8 (2.7, 8.7)	0.25 (0.19–0.34)	19 (9–42)	0.85(0.82–0.88)

**Table 3:** Subgroup analysis of urinary exosomes for DKD diagnosis.

Group	Category	Sensitivity (95% CI)	*P*1	Specificity (95% CI)	*P*2	PLR (95% CI)	NLR (95% CI)	DOR (95% CI)	AUC (95% CI)
Control type	Healthy	0.93 (0.80–0.98)	0.30	0.89 (0.72–0.96)	0.96	8.5 (2.8, 25.5)	0.08 (0.02–0.26)	109 (12–985)	0.96 (0.94–0.98)
	No Healthy	0.83 (0.72–0.90)		0.81 (0.72–0.88)		4.4 (2.7, 7.2)	0.21 (0.12–0.38)	20 (7–57)	0.89 (0.86–0.91)
Biomarker type	miRNA	0.74 (0.69–0.79)	0.00	0.68 (0.59–0.76)	0.00	2.3 (1.8, 3.0)	0.38 (0.30–0.47)	6 (4–10)	0.77 (0.73–0.80)
	protein/polypeptide	0.91 (0.78–0.97)		0.91 (0.85–0.94)		9.7 (5.5, 17.2)	0.09 (0.03–0.27)	103 (21–509)	0.95(0.93–0.97)
Sample size	< 50	0.82 (0.64–0.93)	0.98	0.79 (0.62–0.91)	0.96	4.0 (2.2–7.0)	0.23 (0.11–0.49)	17 (5–54)	0.81 (0.71–0.90)
	≥ 50	0.87 (0.76–0.93)		0.83 (0.74–0.89)		5.1 (3.1–8.6)	0.16 (0.08–0.32)	32 (10–101)	0.91 (0.88–0.93)
Publication year	2011–2018	0.74 (0.67–0.80)	0.08	0.68 (0.60–0.75)	0.01	2.3 (1.7–3.1)	0.38 (0.28–0.51)	6 (3–11)	0.77 (0.73–0.80)
	2019–2025	0.89 (0.78–0.95)		0.86 (0.77–0.92)		6.6 (3.6–12.2)	0.12 (0.05–0.29)	54 (13–223)	0.94 (0.91–0.96)

For both diseases, the subgroups with healthy controls demonstrated higher sensitivity (IgAN: 0.91 vs. 0.69; DKD: 0.93 vs. 0.83) and overall diagnostic performance (DOR, AUC) compared to those with non-healthy controls. In IgAN, this covariate significantly affected specificity (*P*2 = 0.04), with lower specificity observed in the healthy control subgroup. In DKD, this covariate had no significant impact on either sensitivity or specificity (*P*1 = 0.30, *P*2 = 0.96), although numerically superior diagnostic performance was still noted for the healthy control subgroup.

In the subgroup and meta-regression analyses based on biomarker type (miRNA vs. protein/polypeptide), the pooled sensitivity for protein/polypeptide biomarkers was significantly higher than that for miRNA (0.81, 95% CI: 0.64–0.91 vs. 0.76, 95% CI: 0.70–0.82; Regression *P*1 = 0.04). In IgAN, this covariate only affected sensitivity (*P*1 = 0.04), with protein/polypeptide showing higher sensitivity (0.81 vs. 0.76). In DKD, this covariate had a highly significant impact on both sensitivity and specificity (*P*1 ≤ 0.01, *P*2 ≤ 0.01). The sensitivity (0.91 vs. 0.74) and specificity (0.91 vs. 0.68) of protein/polypeptide biomarkers were substantially superior to those of miRNA, suggesting that protein/polypeptide markers possess more stable specificity and sensitivity for DKD diagnosis.

The influence of sample size on diagnostic performance showed no consistent pattern across the two diseases. For IgAN, the small-sample subgroup (< 50 cases) exhibited better diagnostic performance (DOR = 17 vs. 6, AUC = 0.83 vs. 0.76). Conversely, for DKD, the large-sample subgroup (≥ 50 cases) demonstrated superior performance (DOR = 32 vs. 17, AUC = 0.91 vs. 0.81). Heterogeneity tests indicated that all P1 and P2 values were > 0.05, suggesting that sample size was not a primary source of heterogeneity for sensitivity or specificity.

Regarding publication year, studies on IgAN published between 2011–2018 showed higher specificity (0.84 vs. 0.62) and better overall diagnostic performance (DOR = 19 vs. 6, AUC = 0.85 vs. 0.68). For DKD, studies published between 2019–2025 demonstrated comprehensively superior diagnostic performance (sensitivity 0.89 vs. 0.74, specificity 0.86 vs. 0.68, DOR = 54 vs. 6, AUC = 0.94 vs. 0.77). In IgAN, this covariate only affected sensitivity (*P*1 = 0.04); although the numerical sensitivity values were similar between the two periods (0.79 vs. 0.78), a statistically significant difference was present. In DKD, this covariate significantly affected specificity (*P*2 = 0.01) but not sensitivity (*P*1 = 0.08).

#### 3.4.2 Clinical effect

**Figure 10:**
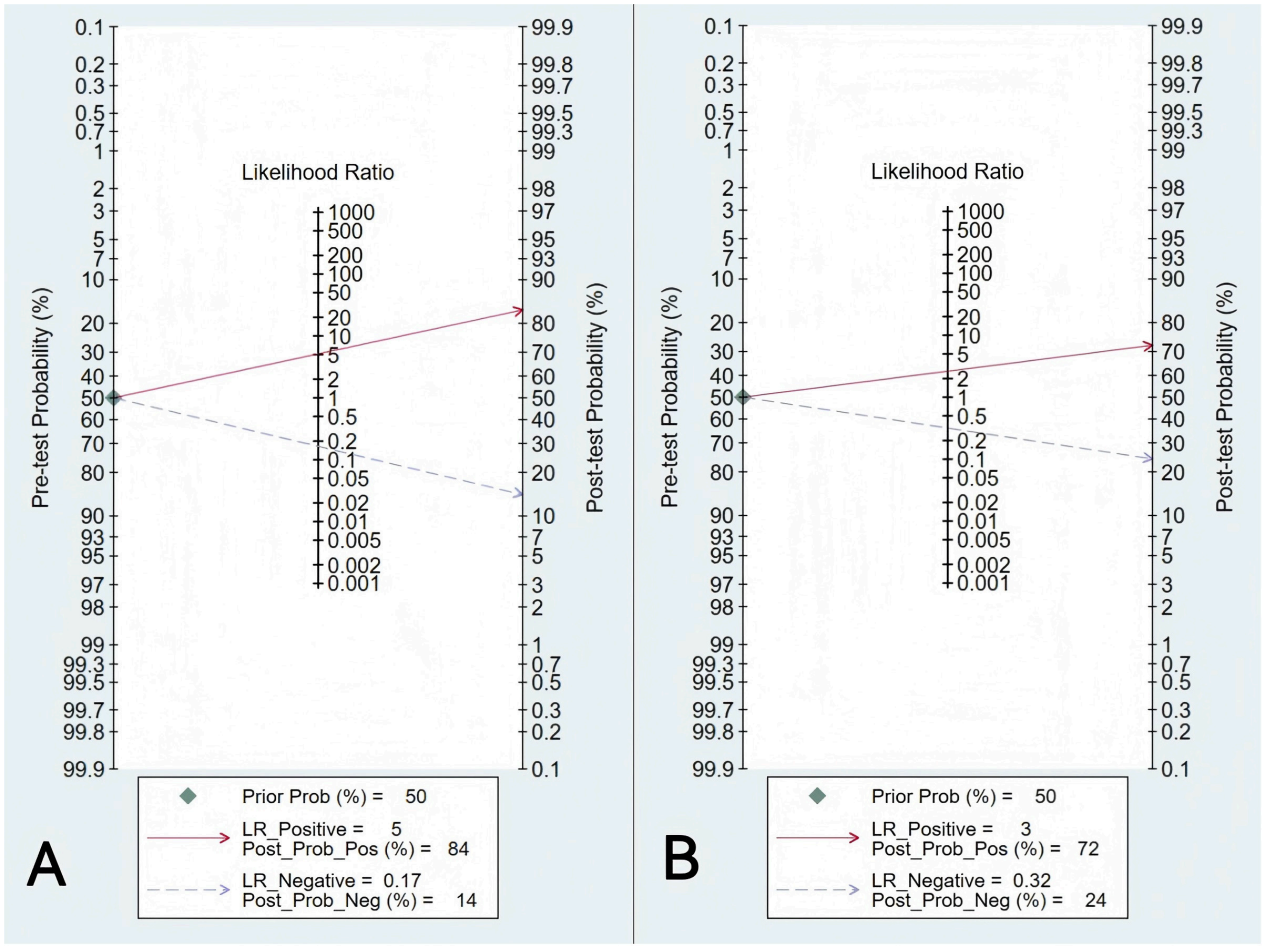
**(A)** Fagan's nomogram for DKD. **(B)** Fagan's nomogram for IgAN.

According to Fagan's nomogram, when the prior probability was set at 50%, the post-test probability after a positive urinary exosome result increased to 84% for DKD, whereas the post-test probability after a negative result was 14%. For IgAN, the corresponding post-test probabilities were 72% after a positive result and 24% after a negative result (Figure 10).

#### 3.4.3 Publication bias

Publication bias among the included studies was investigated using Deeks' funnel plot asymmetry test (Supplementary Figure S1). The results indicated no significant publication bias for either analysis (*P* = 0.27, *P* = 0.12).

## 4 Discussion

A total of 22 studies were included in this meta-analysis. The diagnostic performance of urinary exosomes in two types of kidney disease, namely IgAN and DKD, was systematically evaluated through the synthesis of effect sizes, heterogeneity testing, and sensitivity analysis. The pooled estimates indicate stronger summary diagnostic performance for DKD than for IgAN in published studies. Most included reports used case-control designs, and many also showed frequent unclear risk of bias in patient selection and reference standard.

The finding that urinary exosomes showed measurable diagnostic performance in both diseases is consistent with prior research. For example, a case-control study of IgAN patients reported significantly elevated expression of hsa-miR-451a in urinary exosomes compared to healthy controls, yielding a diagnostic AUC of 0.805 (22), which closely matches the pooled AUC of 0.81 obtained here. Similarly, research on DKD confirmed that urinary exosomal miR-142-3p effectively distinguishes early DKD from healthy individuals with a specificity of 87.2% (13), aligning well with the pooled specificity of 83% reported in this analysis.

The DOR served as a summary measure of diagnostic performance across the included studies. Although DOR values above 10 are often considered suggestive of stronger discriminatory performance, the pooled DOR for IgAN was 8.26 and its 95% confidence interval ranged from 5.03 to 13.56. These results indicate stronger summary discrimination for DKD, whereas the discriminatory performance for IgAN was more moderate (32). This disparity reflects fundamental biological differences between the two pathologies beyond the level of diagnostic metrics. The diagnostic value of urinary exosomes is rooted in their inherent biological properties. As extracellular vesicles secreted by renal cells (33), exosomes transport complex biomolecules, including proteins, lipids, DNA, and RNA (34) that mirror the kidney's physiological and pathological states (7). Moreover, the lipid bilayer membrane of urinary extracellular vesicles, which includes exosomes, shields internal cargo such as proteins and nucleic acids from enzymatic degradation in body fluids (35). This protection confers high stability in urine, providing a crucial material basis for the non-invasive diagnosis of kidney disease.

The differences in the pathological mechanisms of the two diseases directly lead to the divergence in the diagnostic performance of urinary exosomes. The course of DKD is accompanied by progressive decline in renal function and structural kidney damage. In its pathological process, exosomes secreted by renal tubular epithelial cells under high-glucose stimulation can induce renal interstitial fibrosis and extracellular matrix deposition (36). Correspondingly, the expression levels of urinary exosomal biomarkers exhibit broad, continuous dynamic changes in response to the duration of hyperglycemic exposure and the severity of metabolic disorders (21). This may contribute to the higher summary DOR observed for DKD-related urinary exosomes. In contrast, the lower pooled DOR for IgAN and its confidence interval indicate a more limited level of summary discrimination. Urinary exosomes in IgAN may be more relevant as supportive markers for disease differentiation and activity assessment than as stand-alone diagnostic tools. These between-disease differences suggest that the clinical role of urinary exosomes is not identical in DKD and IgAN, with a stronger summary signal in DKD and a more limited auxiliary role in IgAN.

In subgroup analyses for both IgAN and DKD, studies using healthy controls showed higher sensitivity and better summary diagnostic performance than studies using non-healthy controls. This pattern likely reflects wider biological separation between cases and healthy individuals and may magnify diagnostic estimates compared with clinically relevant non-healthy comparator groups. Protein and peptide biomarkers demonstrated significantly better diagnostic performance than miRNA biomarkers for both diseases. The fundamental reason is that proteins and peptides are directly involved in disease pathology, processes such as IgA immune complex deposition and podocyte injury in IgAN, or glomerular basement membrane injury and tubular fibrosis in DKD, are directly mediated by proteins and peptides, whose expression levels thus directly reflect lesion severity. In contrast, miRNAs act as transcriptional regulators that influence disease through indirect modulation of inflammatory and fibrotic pathways, making them more susceptible to interference from other conditions or physiological states and consequently less disease-specific. Heterogeneity testing indicated that this covariate affected only sensitivity in IgAN (*P*1 = 0.04), whereas it exerted a highly significant influence on both sensitivity and specificity in DKD (*P*1 ≤ 0.01, *P*2 ≤ 0.01), suggesting that the expression stability and specificity of protein/peptide biomarkers are more pronounced in DKD. This may relate to specific alterations in protein modifications, such as glycosylation, during DKD progression.

For IgAN diagnosis, studies published between 2011 and 2018 exhibited higher specificity, while for DKD, studies from 2019 to 2025 showed better overall diagnostic performance. This disparity arises from differences in research pathways and stages of technological development for the two diseases. Current IgAN diagnosis still relies on renal biopsy due to the absence of adequately specific and sensitive replacement biomarkers (4). Research has therefore prioritized identifying biomarkers that can distinguish IgAN from other glomerular diseases with high specificity and linking them to IgAN pathology, explaining the earlier emphasis on specificity. For DKD, however, the research objective is early warning. Given its continuous progression and a well-defined pre-clinical window, the goal is to achieve early, non-invasive diagnosis to replace or supplement urinary albumin measurement, driving a focus on developing high-performance diagnostic models and leading to continuous improvements in overall diagnostic efficacy in recent studies. Furthermore, Spearman correlation analysis indicated a significant threshold effect for DKD but not for IgAN. This finding suggests that the included DKD studies used heterogeneous cut-off values, and no common threshold was assumed in the present meta-analysis. Although the bivariate model accommodates part of this between-study variation, the pooled DKD sensitivity and specificity do not correspond to a single clinically applicable threshold. The present DKD results therefore support diagnostic potential at the evidence-synthesis level, but they do not define one threshold for direct clinical use. For IgAN, the absence of a significant threshold effect suggests less threshold-related heterogeneity, although the predominance of case-control designs still limits direct clinical extrapolation.

Early diagnosis benefits patients with IgAN and DKD. While renal biopsy remains the gold standard, its invasive nature and the potential requirement for repeated procedures render it inconvenient and poorly tolerated. Urinary exosome testing may represent a non-invasive research approach with potential future use in adjunctive screening and monitoring. However, the current evidence does not establish routine screening use, particularly for IgAN, where the pooled DOR and Fagan results indicate only a moderate diagnostic gain. Consequently, numerous novel urinary exosomal biomarkers for the early detection of IgAN and DKD have been extensively studied. Leveraging next-generation technologies and other detection methods, urinary exosomes have shown considerable diagnostic potential for these conditions. However, the absence of large-scale cohort studies and a lack of standardized techniques continue to limit their clinical translation. This updated meta-analysis systematically evaluates the diagnostic value of urinary exosomes based on published evidence, aiming to inform the early diagnosis of IgAN and DKD.

This study has several limitations. First, most included studies used case-control designs, which may yield higher diagnostic accuracy estimates than cohort or cross-sectional diagnostic studies. The pooled results therefore reflect published case-control evidence more strongly. Second, DKD showed a significant threshold effect, indicating substantial between-study variability in cut-off values; accordingly, the pooled DKD sensitivity and specificity do not correspond to a single uniform clinical threshold. Third, to maximize the number of eligible studies, we included reports that employed markers with relatively low sensitivity and specificity, which may have affected the stability of the pooled estimates. Fourth, the number of included studies remained limited, and many studies did not report key covariates such as disease stage, mean age, gender, ethnicity, exosome isolation methods, or histopathological characteristics, preventing more detailed analyses of heterogeneity. Fifth, the absence of standardized, unified protocols for isolating, extracting, and detecting urinary exosomes introduced further methodological variability. Finally, restricting the literature search to English-language publications may have introduced language bias.

## 5 Conclusion

This meta-analysis indicates stronger summary diagnostic performance for DKD than for IgAN. For IgAN, the pooled DOR and Fagan results support a moderate adjunctive diagnostic role. Prospective cohort or cross-sectional studies with prespecified cut-offs and clinically relevant comparator groups are needed before urinary exosome biomarkers can be translated into routine use.

## Data Availability

The original contributions presented in the study are included in the article/Supplementary material, further inquiries can be directed to the corresponding author.

## Supplementary material

Supplementary Figure S1 **(A)** Funnel plot for the diagnosis of DKD. **(B)** Funnel plot for the diagnosis of IgAN.
