# Supplementary material for: Diagnostic value of urinary exosomes in patients with IgA nephropathy and diabetic kidney disease: a systematic review and meta-analysis
Source: Front Med (Lausanne). 2026 Jul 23;13:1811135. doi: 10.3389/fmed.2026.1811135 (PMC13444747; doi:10.3389/fmed.2026.1811135)
Supplement: Supplementary file 1 [file Data_Sheet_1.docx]

# Supplementary Text 1 Literature search strategy

**1.Pubmed**

| Search number | Query |
| --- | --- |
| #1 | ("Glomerulonephritis, IGA"[Mesh]) OR "Diabetic Nephropathies"[Mesh] |
| #2 | ((((((((((((((((((((((((((((Berger* Disease*[Title/Abstract]) OR (IGA Glomerulonephritis[Title/Abstract])) OR (IGA Nephropathy[Title/Abstract])) OR (IGA Type Nephritis[Title/Abstract])) OR (Berger* nephropath*[Title/Abstract])) OR (Berger* glomerulonephritis[Title/Abstract])) OR (immunoglobulin A type nephropa[Title/Abstract])) OR (Immunoglobulin A Nephropathy[Title/Abstract])) OR (immunoglobulin A glomeruloneph*[Title/Abstract])) OR (IgA glomerular nephritis[Title/Abstract])) OR (IgA glomerulo-nephritis[Title/Abstract])) OR (IgA nephrotic syndrome[Title/Abstract])) OR (IgA-associated glomerulonephritis[Title/Abstract])) OR (IgA-associated nephropathy[Title/Abstract])) OR (IgA-dominant glomerulonephritis[Title/Abstract])) OR (IgA-induced nephropathy[Title/Abstract])) OR (IgA-related nephropathy[Title/Abstract])) OR (IgAN[Title/Abstract])) OR (Diabetic Nephropath*[Title/Abstract])) OR (Diabetic Kidney Disease*[Title/Abstract])) OR (Diabetic Glomerulosclerosis[Title/Abstract])) OR (Intracapillary Glomerulosclerosis[Title/Abstract])) OR (Kimmelstiel-Wilson Disease*[Title/Abstract])) OR (Kimmelstiel Wilson Disease*[Title/Abstract])) OR (Nodular Glomerulosclerosis[Title/Abstract])) OR (Kimmelstiel-Wilson Syndrome[Title/Abstract])) OR (Kimmelstiel Wilson Syndrome[Title/Abstract])) OR (diabetic renal Disease*[Title/Abstract])) OR (DKD[Title/Abstract]) |
| #3 | (("Glomerulonephritis, IGA"[Mesh]) OR "Diabetic Nephropathies"[Mesh]) OR (((((((((((((((((((((((((((((Berger* Disease*[Title/Abstract]) OR (IGA Glomerulonephritis[Title/Abstract])) OR (IGA Nephropathy[Title/Abstract])) OR (IGA Type Nephritis[Title/Abstract])) OR (Berger* nephropath*[Title/Abstract])) OR (Berger* glomerulonephritis[Title/Abstract])) OR (immunoglobulin A type nephropa[Title/Abstract])) OR (Immunoglobulin A Nephropathy[Title/Abstract])) OR (immunoglobulin A glomeruloneph*[Title/Abstract])) OR (IgA glomerular nephritis[Title/Abstract])) OR (IgA glomerulo-nephritis[Title/Abstract])) OR (IgA nephrotic syndrome[Title/Abstract])) OR (IgA-associated glomerulonephritis[Title/Abstract])) OR (IgA-associated nephropathy[Title/Abstract])) OR (IgA-dominant glomerulonephritis[Title/Abstract])) OR (IgA-induced nephropathy[Title/Abstract])) OR (IgA-related nephropathy[Title/Abstract])) OR (IgAN[Title/Abstract])) OR (Diabetic Nephropath*[Title/Abstract])) OR (Diabetic Kidney Disease*[Title/Abstract])) OR (Diabetic Glomerulosclerosis[Title/Abstract])) OR (Intracapillary Glomerulosclerosis[Title/Abstract])) OR (Kimmelstiel-Wilson Disease*[Title/Abstract])) OR (Kimmelstiel Wilson Disease*[Title/Abstract])) OR (Nodular Glomerulosclerosis[Title/Abstract])) OR (Kimmelstiel-Wilson Syndrome[Title/Abstract])) OR (Kimmelstiel Wilson Syndrome[Title/Abstract])) OR (diabetic renal Disease*[Title/Abstract])) OR (DKD[Title/Abstract])) |
| #4 | "Exosomes"[Mesh] |
| #5 | ((((((((((exosome*[Title/Abstract]) OR (extracellular vesicle*[Title/Abstract])) OR (Secretory Vesicles[Title/Abstract])) OR (microvesicle*[Title/Abstract])) OR (microRNA[Title/Abstract])) OR (miRNA[Title/Abstract])) OR (circRNA[Title/Abstract])) OR (lncRNA[Title/Abstract])) OR (mRNA[Title/Abstract])) OR (protein[Title/Abstract])) OR (proteomic*[Title/Abstract]) |
| #6 | ("Exosomes"[Mesh]) OR (((((((((((exosome*[Title/Abstract]) OR (extracellular vesicle*[Title/Abstract])) OR (Secretory Vesicles[Title/Abstract])) OR (microvesicle*[Title/Abstract])) OR (microRNA[Title/Abstract])) OR (miRNA[Title/Abstract])) OR (circRNA[Title/Abstract])) OR (lncRNA[Title/Abstract])) OR (mRNA[Title/Abstract])) OR (protein[Title/Abstract])) OR (proteomic*[Title/Abstract])) |
| #7 | (urinary[Title/Abstract]) OR (urine[Title/Abstract]) |
| #8 | "Diagnosis"[Mesh] |
| #9 | (((((diagnos*[Title/Abstract]) OR (sensitivity[Title/Abstract])) OR (specificity[Title/Abstract])) OR (accuracy[Title/Abstract])) OR (ROC[Title/Abstract])) OR (AUC[Title/Abstract]) |
| #10 | ("Diagnosis"[Mesh]) OR ((((((diagnos*[Title/Abstract]) OR (sensitivity[Title/Abstract])) OR (specificity[Title/Abstract])) OR (accuracy[Title/Abstract])) OR (ROC[Title/Abstract])) OR (AUC[Title/Abstract])) |
| #11 | ((((("Glomerulonephritis, IGA"[Mesh]) OR "Diabetic Nephropathies"[Mesh]) OR (((((((((((((((((((((((((((((Berger* Disease*[Title/Abstract]) OR (IGA Glomerulonephritis[Title/Abstract])) OR (IGA Nephropathy[Title/Abstract])) OR (IGA Type Nephritis[Title/Abstract])) OR (Berger* nephropath*[Title/Abstract])) OR (Berger* glomerulonephritis[Title/Abstract])) OR (immunoglobulin A type nephropa[Title/Abstract])) OR (Immunoglobulin A Nephropathy[Title/Abstract])) OR (immunoglobulin A glomeruloneph*[Title/Abstract])) OR (IgA glomerular nephritis[Title/Abstract])) OR (IgA glomerulo-nephritis[Title/Abstract])) OR (IgA nephrotic syndrome[Title/Abstract])) OR (IgA-associated glomerulonephritis[Title/Abstract])) OR (IgA-associated nephropathy[Title/Abstract])) OR (IgA-dominant glomerulonephritis[Title/Abstract])) OR (IgA-induced nephropathy[Title/Abstract])) OR (IgA-related nephropathy[Title/Abstract])) OR (IgAN[Title/Abstract])) OR (Diabetic Nephropath*[Title/Abstract])) OR (Diabetic Kidney Disease*[Title/Abstract])) OR (Diabetic Glomerulosclerosis[Title/Abstract])) OR (Intracapillary Glomerulosclerosis[Title/Abstract])) OR (Kimmelstiel-Wilson Disease*[Title/Abstract])) OR (Kimmelstiel Wilson Disease*[Title/Abstract])) OR (Nodular Glomerulosclerosis[Title/Abstract])) OR (Kimmelstiel-Wilson Syndrome[Title/Abstract])) OR (Kimmelstiel Wilson Syndrome[Title/Abstract])) OR (diabetic renal Disease*[Title/Abstract])) OR (DKD[Title/Abstract]))) AND (("Exosomes"[Mesh]) OR (((((((((((exosome*[Title/Abstract]) OR (extracellular vesicle*[Title/Abstract])) OR (Secretory Vesicles[Title/Abstract])) OR (microvesicle*[Title/Abstract])) OR (microRNA[Title/Abstract])) OR (miRNA[Title/Abstract])) OR (circRNA[Title/Abstract])) OR (lncRNA[Title/Abstract])) OR (mRNA[Title/Abstract])) OR (protein[Title/Abstract])) OR (proteomic*[Title/Abstract])))) AND ((urinary[Title/Abstract]) OR (urine[Title/Abstract]))) AND (("Diagnosis"[Mesh]) OR ((((((diagnos*[Title/Abstract]) OR (sensitivity[Title/Abstract])) OR (specificity[Title/Abstract])) OR (accuracy[Title/Abstract])) OR (ROC[Title/Abstract])) OR (AUC[Title/Abstract]))) |

**2.Cochrane**

| Search number | Query |
| --- | --- |
| #1 | MeSH descriptor: [Glomerulonephritis, IGA] explode all trees |
| #2 | MeSH descriptor: [Diabetic Nephropathies] explode all trees |
| #3 | (Berger* Disease*):ti,ab,kw OR (IGA Glomerulonephritis):ti,ab,kw OR (IGA Nephropathy):ti,ab,kw OR (IGA Type Nephritis):ti,ab,kw OR (Berger* nephropath*):ti,ab,kw |
| #4 | (Berger* glomerulonephritis):ti,ab,kw OR (immunoglobulin A type nephropa):ti,ab,kw OR (Immunoglobulin A Nephropathy):ti,ab,kw OR (immunoglobulin A glomeruloneph*):ti,ab,kw OR (IgA glomerular nephritis):ti,ab,kw |
| #5 | (IgA glomerulo-nephritis):ti,ab,kw OR (IgA nephrotic syndrome):ti,ab,kw OR (IgA-associated glomerulonephritis):ti,ab,kw OR (IgA-associated nephropathy):ti,ab,kw OR (IgA-dominant glomerulonephritis):ti,ab,kw |
| #6 | (IgA-induced nephropathy):ti,ab,kw OR (IgA-related nephropathy):ti,ab,kw OR (IgAN):ti,ab,kw OR (Diabetic Nephropath*):ti,ab,kw OR (Diabetic Kidney Disease*):ti,ab,kw |
| #7 | (Diabetic Glomerulosclerosis):ti,ab,kw OR (Intracapillary Glomerulosclerosis):ti,ab,kw OR (Kimmelstiel-Wilson Disease*):ti,ab,kw OR (Kimmelstiel Wilson Disease*):ti,ab,kw OR (Nodular Glomerulosclerosis):ti,ab,kw |
| #8 | (Kimmelstiel-Wilson Syndrome):ti,ab,kw OR (Kimmelstiel Wilson Syndrome):ti,ab,kw OR (diabetic renal Disease*):ti,ab,kw OR (DKD):ti,ab,kw |
| #9 | #1 or #2 or #3 or #4 or #5 or #6 or #7 |
| #10 | MeSH descriptor: [Exosomes] explode all trees |
| #11 | (exosome*):ti,ab,kw OR (extracellular vesicle*):ti,ab,kw OR (Secretory Vesicles):ti,ab,kw OR (microvesicle*):ti,ab,kw OR (microRNA):ti,ab,kw |
| #12 | (miRNA):ti,ab,kw OR (circRNA):ti,ab,kw OR (lncRNA):ti,ab,kw OR (mRNA):ti,ab,kw OR (protein):ti,ab,kw |
| #13 | (proteomic*):ti,ab,kw |
| #14 | #10 or #11 or #12 or #13 |
| #15 | (urinary):ti,ab,kw OR (urine):ti,ab,kw |
| #16 | MeSH descriptor: [Diagnosis] explode all trees |
| #17 | (diagnos*):ti,ab,kw OR (sensitivity):ti,ab,kw OR (specificity):ti,ab,kw OR (accuracy):ti,ab,kw OR (ROC):ti,ab,kw |
| #18 | (AUC):ti,ab,kw |
| #19 | #16 or #17 or #18 |
| #20 | #9 and #14 and #15 and #19 |

**3.Embase**

| Search number | Query |
| --- | --- |
| #1 | 'immunoglobulin a nephropathy'/exp |
| #2 | 'diabetic nephropathy'/exp |
| #3 | 'berger* disease*':ab,ti OR 'iga glomerulonephritis':ab,ti OR 'iga nephropathy':ab,ti OR 'iga type nephritis':ab,ti OR 'berger* nephropath*':ab,ti OR 'berger* glomerulonephritis':ab,ti OR 'immunoglobulin a type nephropa':ab,ti OR 'immunoglobulin a nephropathy':ab,ti OR 'immunoglobulin a glomeruloneph*':ab,ti OR 'iga glomerular nephritis':ab,ti OR 'iga glomerulo-nephritis':ab,ti OR 'iga nephrotic syndrome':ab,ti OR 'iga-associated glomerulonephritis':ab,ti OR 'iga-associated nephropathy':ab,ti OR 'iga-dominant glomerulonephritis':ab,ti OR 'iga-induced nephropathy':ab,ti OR 'iga-related nephropathy':ab,ti OR igan:ab,ti OR 'diabetic nephropath*':ab,ti OR 'diabetic kidney disease*':ab,ti OR 'diabetic glomerulosclerosis':ab,ti OR 'intracapillary glomerulosclerosis':ab,ti OR 'kimmelstiel-wilson disease*':ab,ti OR 'kimmelstiel wilson disease*':ab,ti OR 'nodular glomerulosclerosis':ab,ti OR 'kimmelstiel-wilson syndrome':ab,ti OR 'kimmelstiel wilson syndrome':ab,ti OR 'diabetic renal disease*':ab,ti OR dkd:ab,ti |
| #4 | #1 OR #2 OR #3 |
| #5 | 'exosome'/exp |
| #6 | exosome*:ab,ti OR 'extracellular vesicle*':ab,ti OR 'secretory vesicles':ab,ti OR microvesicle*:ab,ti OR microrna:ab,ti OR mirna:ab,ti OR circrna:ab,ti OR lncrna:ab,ti OR mrna:ab,ti OR protein:ab,ti OR proteomic*:ab,ti |
| #7 | #5 OR #6 |
| #8 | urinary:ab,ti OR urine:ab,ti |
| #9 | 'diagnosis'/exp |
| #10 | diagnos*:ab,ti OR sensitivity:ab,ti OR specificity:ab,ti OR accuracy:ab,ti OR roc:ab,ti OR auc:ab,ti |
| #11 | #9 OR #10 |
| #12 | #4 AND #7 AND #8 AND #11 |

**4.Web of science**

| Search number | Query |
| --- | --- |
| #1 | "Berger* Disease* (Topic) OR IGA Glomerulonephritis (Topic) OR IGA Nephropathy (Topic) OR IGA Type Nephritis (Topic) OR Berger* nephropath* (Topic) OR Berger* glomerulonephritis (Topic) OR immunoglobulin A type nephropa (Topic) OR Immunoglobulin A Nephropathy (Topic) OR immunoglobulin A glomeruloneph* (Topic) OR IgA glomerular nephritis (Topic) OR IgA glomerulo-nephritis (Topic) OR IgA nephrotic syndrome (Topic) OR IgA-associated glomerulonephritis (Topic) OR IgA-associated nephropathy (Topic) OR IgA-dominant glomerulonephritis (Topic) OR IgA-induced nephropathy (Topic) OR IgA-related nephropathy (Topic) OR IgAN (Topic) OR Diabetic Nephropath* (Topic) OR Diabetic Kidney Disease* (Topic) OR Diabetic Glomerulosclerosis (Topic) OR Intracapillary Glomerulosclerosis (Topic) OR Kimmelstiel-Wilson Disease* (Topic) OR Kimmelstiel Wilson Disease* (Topic) OR Nodular Glomerulosclerosis (Topic) OR Kimmelstiel-Wilson Syndrome (Topic) OR Kimmelstiel Wilson Syndrome (Topic) OR diabetic renal Disease* (Topic) OR DKD (Topic) " |
| #2 | "exosome* (Topic) OR extracellular vesicle* (Topic) OR Secretory Vesicles (Topic) OR microvesicle* (Topic) OR microRNA (Topic) OR miRNA (Topic) OR circRNA (Topic) OR lncRNA (Topic) OR mRNA (Topic) OR protein (Topic) OR proteomic* (Topic) " |
| #3 | "urinary (Topic) OR urine (Topic) " |
| #4 | "diagnos* (Topic) OR sensitivity (Topic) OR specificity (Topic) OR accuracy (Topic) OR ROC (Topic) OR AUC (Topic) ,Timespan: All years (Publication Date) " |
| #5 | "#1 AND #2 AND #3 AND #4 " |
